# The AtMYB60 transcription factor regulates stomatal opening by modulating oxylipin synthesis in guard cells

**DOI:** 10.1038/s41598-021-04433-y

**Published:** 2022-01-11

**Authors:** Fabio Simeoni, Aleksandra Skirycz, Laura Simoni, Giulia Castorina, Leonardo Perez de Souza, Alisdair R. Fernie, Saleh Alseekh, Patrick Giavalisco, Lucio Conti, Chiara Tonelli, Massimo Galbiati

**Affiliations:** 1 Dipartimento di Bioscienze, Università degli Studi di Milano, Milan, Italy; 2 Boyce Thompson Institute, Ithaca, NY USA; 3 Dipartimento di Scienze Agrarie e Ambientali-Produzione, Territorio, Agroenergia, Università degli Studi di Milano, Milan, Italy; 4 Max Planck Institute of Molecular Plant Physiology, Potsdam-Golm, Germany; 5 Center for Plant Systems Biology and Biotechnology, Plovdiv, Bulgaria; 6 Metabolomics Core Facility, Max Planck Institute for Biology of Ageing, Cologne, Germany; 7 Istituto di Biologia e Biotecnologia Agraria, Consiglio Nazionale Delle Ricerche, Milan, Italy

**Keywords:** Plant hormones, Jasmonic acid, Plant sciences, Plant physiology

## Abstract

Stomata are epidermal pores formed by pairs of specialized guard cells, which regulate gas exchanges between the plant and the atmosphere. Modulation of transcription has emerged as an important level of regulation of stomatal activity. The AtMYB60 transcription factor was previously identified as a positive regulator of stomatal opening, although the details of its function remain unknown. Here, we propose a role for AtMYB60 as a negative modulator of oxylipins synthesis in stomata. The *atmyb60-1* mutant shows reduced stomatal opening and accumulates increased levels of 12-oxo-phytodienoic acid (12-OPDA), jasmonic acid (JA) and jasmonoyl-l-isoleucine (JA-Ile) in guard cells. We provide evidence that 12-OPDA triggers stomatal closure independently of JA and cooperatively with abscisic acid (ABA) in *atmyb60-1*. Our study highlights the relevance of oxylipins metabolism in stomatal regulation and indicates *AtMYB60* as transcriptional integrator of ABA and oxylipins responses in guard cells.

## Introduction

Several of the manifold interactions between plants and their surrounding environment are modulated by stomata^1^. Stomatal regulation is a “minute-by-minute decisional process” through which guard cells integrate external stimuli and endogenous signals to adjust the opening of the pore to the prevailing environmental conditions. Tuning of stomatal aperture relies on the coordination of a complex network of signaling pathways, mostly activated by plant hormones^2^. Among them, ABA is well-known as the main regulator of stomatal closure in response to water stress. Additional positive regulators of stomatal closure include jasmonates (JAs) and salicylic acid (SA)^2^.

JAs are oxylipin-derived hormones involved in several physiological processes, ranging from the regulation of development and fertility, to defense responses, stress adaptation and stomatal activity^3^. Oxylipins biosynthesis is mediated by lipoxygenases (LOXs), which catalyze the oxygenation of C16 or C18 fatty acids at either the C9 (9-LOXs) or C13 position (13-LOXs). 13-LOX-derived hydroperoxy products include 12-OPDA, JA, JA-Ile and methyl-jasmonate (Me-JA)^4^. Increasing evidence indicates that 12-OPDA is an active signaling molecule, besides being a JA metabolic intermediate. Several physiological and developmental processes are regulated by overlapping activities of 12-OPDA and JA. In addition, 12-OPDA modulates specific plant responses independently of JA, including gene expression and stomatal activity^5–8^.

Transcription factors (TF) are key regulatory hubs that control hormone homeostasis and hormone-induced responses. Cell-specific transcriptomic analyses and genetic screens uncovered guard cell-related TFs regulating different aspects of stomatal activity^9^. Among them, AtMYB60, belonging to the large R2R3 MYB subfamily, has been implicated in light-induced stomatal opening^10^. *AtMYB60* is expressed in guard cells under optimal growth conditions, whereas its transcript abundance rapidly declines following exposure to drought or ABA^10–13^. Loss of *AtMYB60* function results in constitutively reduced stomatal opening and increased drought resistance^10^. Given the relevance of the hormonal regulation of stomatal activity, we were particularly interested in understanding if the *atmyb60-1* mutation altered the accumulation of stomatal-closure promoting hormones in guard cells. Here we report the function of *AtMYB60* as a negative modulator of oxylipins synthesis in stomata. The cellular specificity of the *AtMYB60* regulation provides a new level of regulation, allowing the precise spatial control of oxylipin-mediated responses.

## Results

### *AtMYB60* negatively modulates the accumulation of JAs and 12-OPDA in guard cells

**Figure 1:**
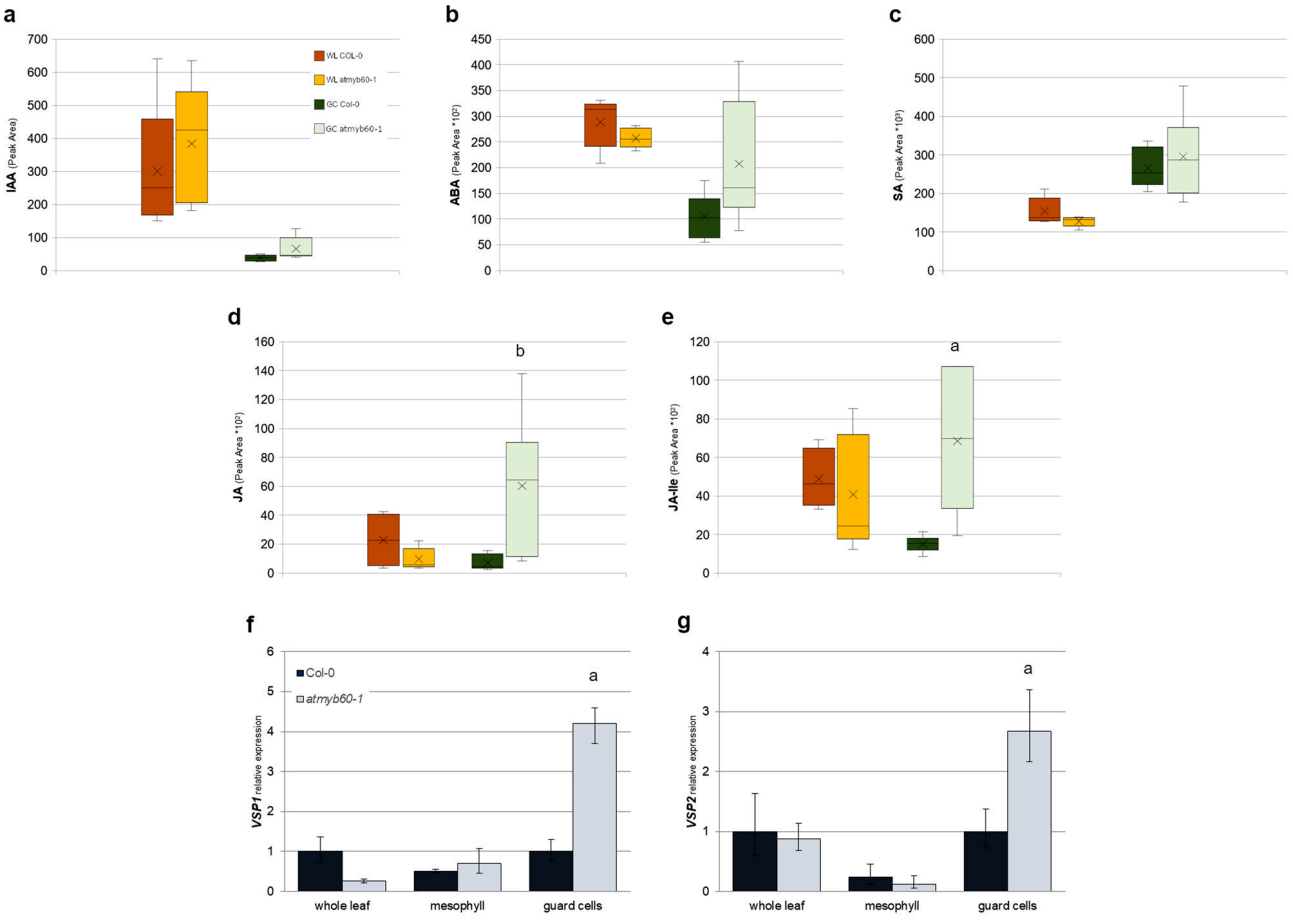
Guard cells from the *atmyb60*-1 mutant over-accumulate JA and JA-Ile. (**a**–**d**) quantitative analysis of IAA (**a**), ABA (**b**), SA (**c**), JA (**d**) and JA-Ile (**e**) in whole leaves (WL), or epidermal fractions enriched in guard cells (GC). (**f**–**g**), qPCR analysis of *Vsp1* (**f**) and *Vsp2* (**g**) expression in whole leaves and laser-microdissected mesophyll or guard cells. Relative gene expression was normalized to the expression of the *AtACTIN2* gene. “a” and “b” indicates significant differences between wild type and *atmyb60-1* samples at *P* < 0.01 and *P* < 0.05, respectively (*t*-test).

First, we compared the level of ABA, SA, JA and JA-Ile in guard cells isolated from wild type and *atmyb60-1* plants by the leaf blending method^14^ (Supplementary Fig. 1a, b). Quantification of the auxin indolacetic acid (IAA), indirectly involved in stomatal opening, was also included as a negative control. IAA was at invariant levels between wild type and *atmyb60-1* samples, although reduced in guard cells compared with intact leaves (Fig. 1a). Similarly, ABA content was diminished in guard cells. *atmyb60-1* stomata showed great variability in ABA accumulation, although they did not reveal significant differences compared with the wild type (Fig. 1b). Consistent with data from hormone quantification, expression of selected ABA-responsive genes was reduced in the guard cell-fractions compared with whole leaves and did not disclose differences between wild type and mutant samples (Supplementary Fig. 2). SA levels were increased in the guard cell-enriched fraction, regardless of the genetic background (Fig. 1c). Most interestingly, accumulation of both JA and JA-Ile was augmented in guard cells from the *atmyb60-1* mutant compared with the wild type (Fig. 1d,e). However, no differences were observed in the relative content of JA and JA-Ile in whole leaves from the two genotypes. Expression of the JA-induced genes *Vegetative Storage Protein 1* (*VSP1*) and *2* (*VSP2*)^15^ was enhanced in guard cells laser-microdissected (LM) from the mutant compared with the wild type. Conversely, no change was visible in LM-purified mesophyll cells or in intact leaves (Fig. 1f,g). This provided further support to the finding that *atmyb60-1* accumulates increased level of JAs in stomata and led us to hypothesize that: (i) *AtMYB60* negatively regulates the biosynthesis of JAs in guard cells and that, (ii) the overaccumulation of JAs, or related oxylipins, contributes to reducing stomatal opening in the *atmyb60-1* mutant.

**Figure 2:**
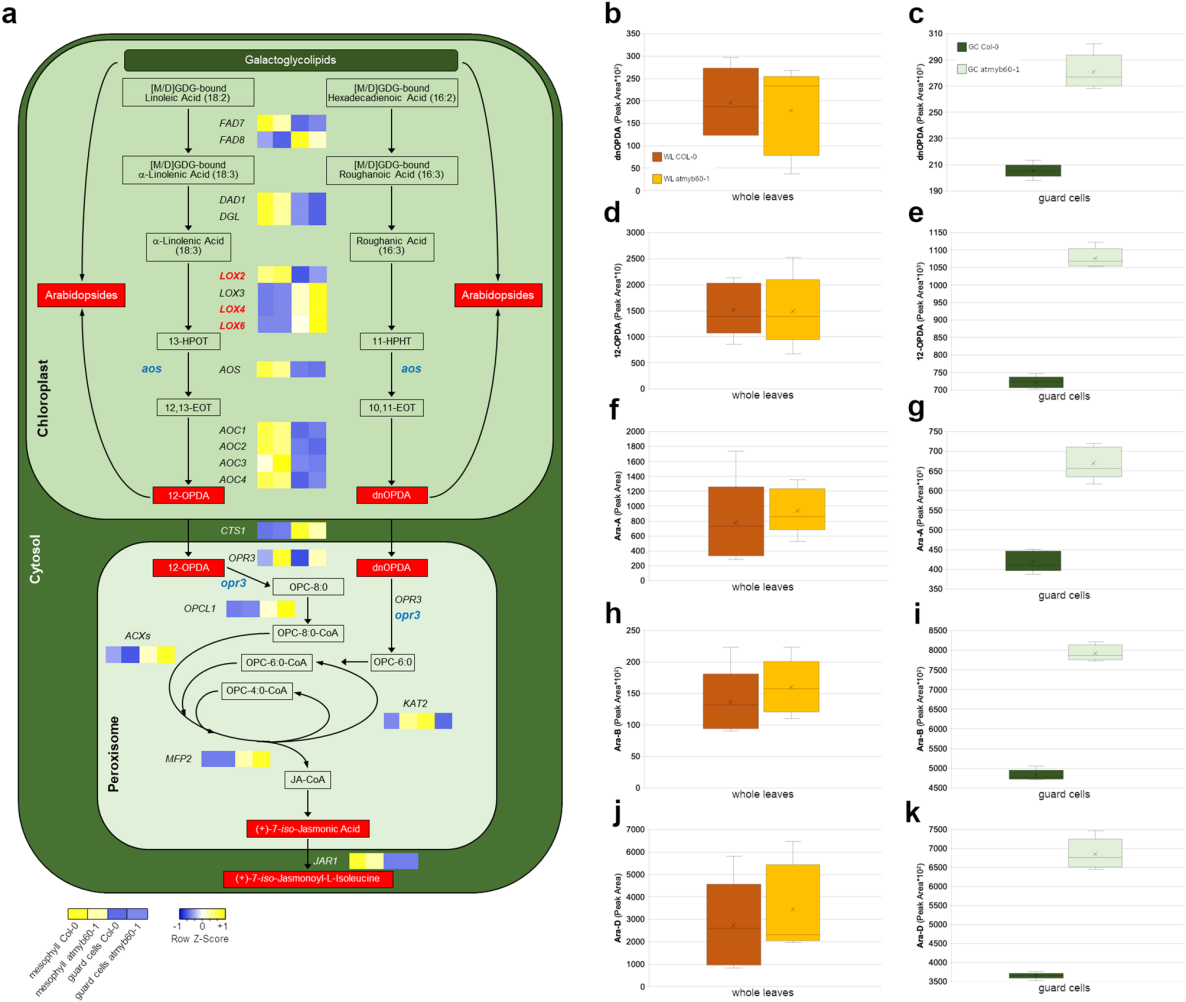
Accumulation of free and esterified 12-OPDA and dnOPDA is enhanced in guard cells from the *atmyb60-1* mutant. (**a**) Schematic representation of the JA biosynthetic pathway. Red boxes represent metabolites identified as over-accumulated in the *atmyb60-1* mutant compared with the wild type. Genes coding for JA biosynthetic enzymes are indicated by their acronyms (uppercase, italics). Heatmaps indicate relative gene expression in LM-purified Col-0 or *atmyb60-1* mesophyll and guard cells (Heatmapper, http://www.heatmapper.ca). Genes upregulated in the mutant as compared with the wild type are highlighted in red (*13-LOXs*). Mutant alleles employed in the study are highlighted in blue (lowercase, italics). Modified from Acosta and Farmer (2010)^4^. Abbreviations:[M/D]GDG, mono- or di-galactosyldiacylglycerol; 13-HPOT, 13(S)-hydroperoxy-octadecatrienoic acid; 12,13-EOT, (13S)-12,13-epoxy-octadecatrienoic acid; OPC-8:0, 3-oxo-2(2′-pentenyl)-cyclopentane-1-octanoic acid; OPC-6:0, 3-oxo-2(2′-pentenyl)-cyclopentane-1-hexanoic acid; OPC4:0, 3-oxo-2(2*'-*pentenyl)-cyclopentane-1-butanoic acid; CoA, Coenzyme A. (**b**–**k**), relative amounts of free dnOPDA (**b**,**c**), free 12-OPDA (**d**,**e**), Ara-A (**f**,**g**), Ara-B (**h**,**i**) and Ara-D (**j**,**k**), in whole leaves or epidermal fractions enriched in guard cells obtained from wild type or *atmyb60-1* plants. Note the different scale of the Y-axis between whole leaves and guard cells. "a" indicates significant differences between wild type and *atmyb60-1* samples (P < 0.01, *t*-test).

A key step in JA synthesis is the production of dinor-OPDA (dnOPDA) and 12-OPDA, mediated by 13-LOXs (Fig. 2a). In addition to being a JA precursor, 12-OPDA is a signaling molecule which activates a JA-independent signaling leading to stomatal closure^6^. We uncovered significantly higher levels of 12-OPDA (and dnOPDA) in guard cells from the *atmyb60-1* mutant compared with the wild type (Fig. 2b–e). In Arabidopsis, the majority of 12-OPDA and dnOPDA is esterified to MGDG or DGDG, to produce Arabidopsides (Ara) (Fig. 2a). 12-OPDA and Ara are present at very low level in plants grown under standard condition, whereas they rapidly accumulate following leaf wounding^16^. Consistently, the mechanical disruption of plant tissues, employed to purify the stomata-enriched fraction, resulted in a drastic increase in the amount of 12-OPDA and Arabidopsides in guard cells compared with intact leaves, in both the wild type and the mutant. Nevertheless, guard cells from *atmyb60-1* showed significantly higher levels of Ara-A, -B and -D relatively to stomata isolated from the wild type (Fig. 2f–k).

The overaccumulation of both free and esterified dnOPDA and 12-OPDA in *atmyb60-1* guard cells is consistent with the increased levels of JA and JA-Ile (Fig. 1d,e) and advocates the possible involvement of 12-OPDA in reducing stomatal opening in the mutant.

### Expression of *13-LOXs* is upregulated in *atmyb60-1* guard cells

We next assessed the expression of oxylipins biosynthetic genes in wild type and *atmyb60-1* LM-purified mesophyll and guard cells to gain more insight into the genes and the metabolic steps possibly modulated by *AtMYB60* (Fig. 2a). Expression of *FATTY ACID DESATURASE7* and *-8* (*FAD7*, *-8*) and of the lipases *DEFECTIVE IN ANTHER DEHISCENCE1* (*DAD1*) and *DONGLE* (*DGL*), involved in the initial plastidial steps, did not reveal substantial variations between wild type and mutant tissues (Fig. 2a, Supplementary Fig. 3a–d).

**Figure 3:**
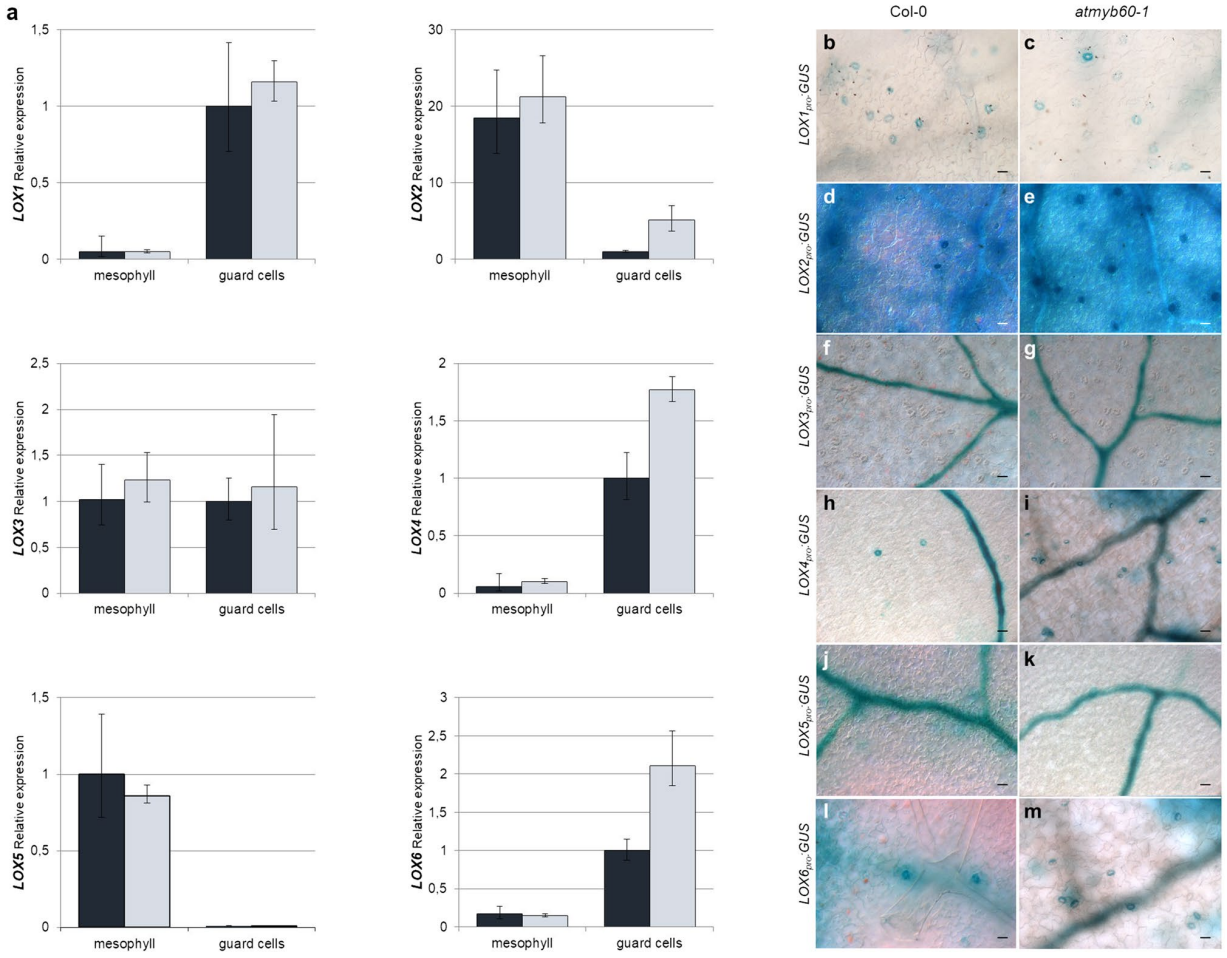
*AtMYB60* negatively regulates expression of *13-LOX* genes. (**a**) qPCR analysis of *9-LOXs* (*LOX1* and *LOX5*) and *13 LOXs* (*LOX2*, *LOX3*, *LOX4* and *LOX6*) expression in mesophyll or guard cells laser-microdissected from wild type or *atmyb60-1* leaves. Relative gene expression was normalized to the expression of the *AtACTIN2* gene. (**b**–**m**) Histochemical analysis of GUS activity in Arabidopsis stable transgenic *LOXpro*:*GUS* lines in the wild type (Col-0) or *atmyb60-1* background. Developing leaves from 15-day-old plants were incubated in the staining solution for 24 h. Scale bars represent 20 µm.

Interestingly, expression of the lipoxygenase genes *LOX2*, *LOX4* and *LOX6*, was slightly but significantly upregulated in *atmyb60-1* stomata compared with the wild type (Figs. 2a, 3a). The Arabidopsis genome contains six LOX isoforms, grouped in 9-LOXs (LOX1 and LOX5) and 13-LOXs (LOX2, LOX3, LOX4, and LOX6). The latter are involved in the plastidial oxygenation of 18:3 and 16:3 acids, whereas 9-LOXs are localized outside of the plastid and do not contribute to 12-OPDA and JA production. *LOX1* and *LOX6* have been previously identified as guard cell-related genes^17, 18^. Our analysis confirmed the cellular specificity of *LOX1* and *LOX6* and revealed the preferential expression of *LOX4* in stomata. *LOX2*, although primarily expressed in the mesophyll, was highly expressed in stomata. Consistently with the cellular specificity of *AtMYB60*, we observed upregulation of *LOX2*, *LOX4* and *LOX6*, in *atmyb60-1* guard cells but not in mesophyll cells (Fig. 3a). Progressing along the pathway, expression of *ALLENE OXIDE SYNTHASE* (*AOS*) and of the four *ALLENE OXIDE CYCLASE* (*AOC*) genes, mediating the conversion of the LOX-derived products to 12-OPDA and dnOPDA, did not reveal variations between wild type and mutant tissues (Fig. 2a, Supplementary Fig. 3e–i). Expression of *COMATOSE1* (*CTS1*) and *OPDA- REDUCTASE3* (*OPR3*), involved in the transport of 12-OPDA and dnOPDA to the peroxisome and in their successive reduction to OPC-8:0 and OPC-6:0, did not show significant differences among mesophyll and guard cells from wild type or *atmyb60-1* leaves (Fig. 2a, Supplementary Fig. 3j,k). Likewise, the genes involved in the final β-oxidation of OPC-8:0 to produce JA, including *OPC-8:0 CoA LIGASE1* (*OPCL1*), *ACYL-CoA OXIDASE1* and -*5* (*ACX1*, -*5*), *MULTIFUNCTIONAL PROTEIN2* (*MFP2*), and *L-3-KETOACYL CoA THIOLASE2* (*KAT2*) were invariant in their expression between the two genotypes (Fig. 2a, Supplementary Fig. 3l–p). Finally, expression of *JASMONATE RESISTANT1* (*JAR1*), which catalyzes the conversion of JA to the biologically active JA-Ile, although drastically reduced in guard cells compared with the mesophyll, did not show differences between the wild type and the *atmyb60-1* mutant (Fig. 2a, Supplementary Fig. 3q).

Overall, our analysis specifically uncovered differences in the expression of *13-LOXs*, which was generally upregulated in *atmyb60-1* guard cells, compared with the wild type. This finding provides further support to the role of *AtMYB60* as a negative regulator of the oxylipins biosynthetic pathway in stomata, and specifies *LOX2*, *LOX4* and *LOX6* as possible targets.

### *AtMYB60* negatively regulates *13-LOXs* expression

We employed a transient expression assay in tobacco to provide a first hint for addressing the function of *AtMYB60* in regulating *13-LOXs* expression. Leaves of *N. benthamiana* were infiltrated with individual *LOXpromoter:GUS* fusions along with a control *CaMV35S* empty vector or with the *CaMV35S:AtMYB60* construct, overexpressing the MYB60 protein. Co-infiltration with *CaMV35S:AtMYB60* significantly reduced the activity of the *13-LOX* promoters previously found to be upregulated in *atmyb60-1* mutant. Conversely, co-expression with the AtMYB60 protein did not affect the activity of the two 9-LOX promoters (Supplementary Fig. 4a).

Next, we produced wild type (Col-0) and *atmyb60-1* stable transgenic lines carrying the *LOX*_*pro*_*:GUS* fusions to compare activities of the *LOX* promoters in the two genetic backgrounds. Overall, the wild type *LOX*_*pro*_*:GUS* lines revealed patterns of GUS activity comparable with the tissue localization described in previous studies^19, 20^ (Supplementary Fig. 4b-g). Noteworthy preceding works did not report activity in stomata for any of the six *LOX* promoters, despite the preferential expression of *LOX1*, *LOX4* and *LOX6* in guard cells, as revealed by the aforementioned qPCR or gene-chip studies^17, 18^. We performed a kinetic staining analysis to assess the level of GUS activity in the different *LOX*_*pro*_*:GUS* lines. Consistent with previous studies, we did not detect GUS activity in stomata from any of the reporter lines following up to 14 h of incubation in the staining solution (Supplementary Fig. 5). Only after prolonged GUS staining (up to 24 h), were *LOX1*, *LOX2*, *LOX4* and *LOX6* promoter activities detected in guard cells albeit this was limited to a small number of stomata (Fig. 3b,d,h,l). This finding suggests that when present, activity of the *LOX* promoters in guard cells was very low with GUS expression barely reaching the threshold of detection. Introgression of the transgenes in the *atmyb60-1* mutant did not produce detectable changes in the activity of the *LOX1*, *LOX3* and *LOX5* promoters in guard cells (Fig. 3c,g,k). Despite the high variability in GUS expression across lines and individual leaves, we consistently observed an increased number of GUS-positive stomata in the *atmyb60-1 LOX2*-*, LOX4*- and *LOX6*_*pro*_*:GUS* lines as compared with the respective Col-0 lines (Fig. 3e,i,m, Supplementary Table 1). Taken together, analyses of the *LOX*_*pro*_*:GUS* transgenics were in accordance with results from the transient activation experiment in *N. benthamiana* and corroborated the involvement of *AtMYB60* in negatively regulating the expression of *LOX2*, *LOX4* and *LOX6* in guard cells.

### Reduced stomatal opening in *atmyb60-1* is associated with 12-OPDA accumulation in guard cells

**Figure 4:**
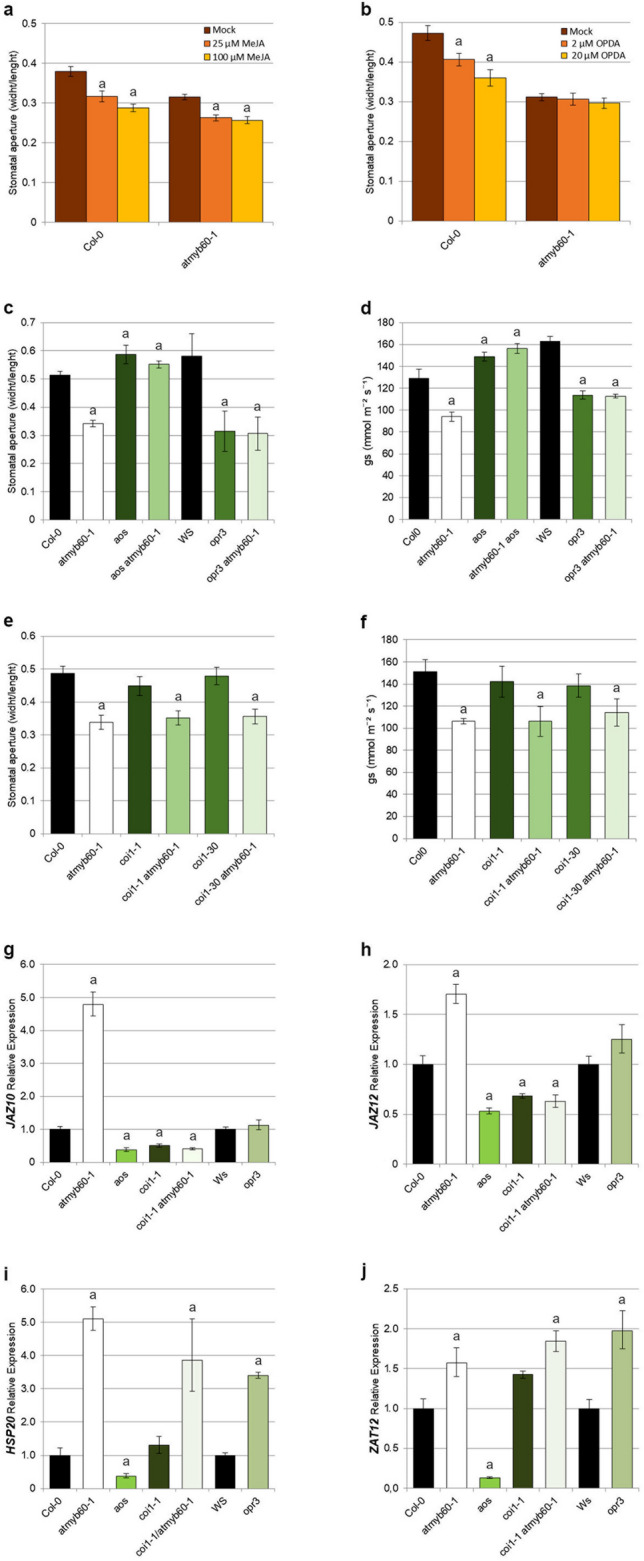
Defective stomatal opening in *atmyb60-1* is associated with 12-OPDA accumulation in guard cells. (**a**,**b**), Stomatal aperture measurements in Col-0 and *atmyb60-1* epidermal strips determined after 4 h of treatment with 25 or 100 μM MeJA (**a**) and with 2 or 20 μM 12-OPDA (**b**). Stomatal opening is expressed as the width/length ratio of the stomatal pore. (**c**,**d**) Stomatal aperture (**c**) and stomatal conductance (*g*_*s*_) (**d**) of Col-0, *atmyb60-1*, *aos*, *aos* *atmyb60-1*, Ws, *opr3* and *opr3 atmyb60-1* plants. (**e**,**f**) stomatal aperture (**e**) and stomatal conductance (**f**) of Col-0, *atmyb60-1,* *coi1-1*, *coi1-1* *atmyb60-1*, *coi1-30* and *coi1-30 atmyb60-1* leaves. Stomatal opening values represent the mean ± standard error of three separate experiments (n = 100–150 stomata per genotype per experiment). Stomatal conductance values represent the mean ± standard error of three separate experiments (n = 10 leaves per genotype per experiment). (**g**–**j**) qPCR analysis of *JAZ10* (**g**), *JAZ12* (**h**), *HSP20* and *ZAT12* (**j**) expression in Col-0, *atmyb60-1*, *aos*, *coi1-1*, *coi1-1* *atmyb60-1*, Ws and *opr3* 10-day-old seedlings. Relative gene expression was normalized to the expression of the *AtACTIN2* gene. "a" indicates statistically significant difference between mutants and their respective wild type (Col-0 or Ws) (P < 0.01, *t*-test).

JA and its precursor 12-OPDA have been shown to play distinct and independent roles in promoting stomatal closure^6, 7^. As *atmyb60-1* guard cells accumulated increased level of both JA and 12-OPDA, we sought to assess the relative contribution of each molecule to the stomatal defects depicted by the mutant. Treatment of epidermal peels with increasing doses of MeJA induced comparable stomatal closure in wild type and *atmyb60-1* stomata (Fig. 4a), whereas application of 12-OPDA triggered stomatal closure in the wild type but not in *atmyb60-1* peels (Fig. 4b).

We next employed a genetic approach using the *aos* and *opr3-1* biosynthetic mutants, impairing activity of *AOS* and of *OPR3*, respectively^6^ (Fig. 2a). Homozygous *aos* plants are devoid of both 12-OPDA and JA, and show increased stomatal opening compared with the wild type. By contrast, *opr3-1* plants, which only retain residual level of JA and over-accumulate 12-OPDA, disclosed constitutively enhanced closure of the stomatal pore^6^. The analysis of stomatal opening and conductance (*g*_*s*_) in the *atmyb60-1 aos* and *atmyb60-1 opr3-1* double mutants revealed that *aos* was epistatic to *atmyb60-1* (Fig. 4c,d). This suggests that the combined deficiency of 12-OPDA and JA in the *atmyb60-1 aos* background could rescue the negative effect of the *atmyb60-1* mutation on stomatal opening. The *atmyb60-1 opr3-1* double mutant did not show additive effects, as stomatal opening and conductance were similar to those of the single mutants (Fig. 4c,d).

It is important to consider that, *opr3-1* is not a complete null allele, as it accumulates minimal amounts of JA^21^ and that an OPR3-independent pathway has been recently demonstrated for JA synthesis^22^. We cannot exclude that the reduced stomatal opening in the *atmyb60-1 opr3-1* results from residual JA activity in this background.

To overcome this limitation, we exploited the JA-insensitive *coi1-1* and *coi1-30* mutations, impairing activity of the JA receptor CORONATINE INSENSITIVE1 (COI1)^23, 24^. COI1 plays a central role in JA signaling and *coi1* mutants are defective in all JA-dependent responses^25^. Interestingly, the loss of the *COI1* function did not rescue the *atmyb60-1* stomatal defect in the double *atmyb60-1 coi1-1* and *atmyb60-1 coi1-30* mutants (Fig. 4e,f). Given that JA signaling is completely abolished in these backgrounds, we reasoned that the reduced stomatal opening in the *atmyb60-1 coi1-1* and *atmyb60-1 coi1-30* double mutants was independent of JA signaling. This suggests that the over-accumulation of 12-OPDA, associated with the *atmyb60-1* mutation, was sufficient to reduce stomatal opening in *atmyb60-1 coi1-1* and *atmyb60-1 coi1-30*.

An alternative explanation for the presumed role of 12-OPDA in reducing stomatal opening in *atmyb60-1* is the possible impairment of JA signaling resulting from the loss of the *AtMYB60* function. The reported upregulation of *VSP1* and *-2* in guard cells from the mutant (Fig. 1f,g) and the responsiveness of *atmyb60-1* stomata to exogenous Me-JA (Fig. 4a) argue against this possibility. Furthermore, expression of *JAsmonate Zim domain10* (*JAZ10*) and *JAZ12*, requiring the presence of JA and of an intact COI1-dependent signaling pathway^26^, was significantly upregulated in *atmyb60-1* (Fig. 4g,h). This result is consistent with the overaccumulation of JA in *atmyb60-1* and suggests the presence of an intact JA-signaling network in this background. In the *opr3-1* mutant, *JAZ10* and *JAZ12* expression was activated to the same extent as in the wild type (Ws), which is conceivable with the presence of residual amounts of JA in this allele. Conversely, expression of the 12-OPDA-responsive genes *Heat Shock Protein20* (*HSP20*) and *Zinc Finger of Arabidopsis Thaliana12* (*ZAT12*)^27^ was drastically reduced in the 12-OPDA-deficient *aos* mutant and upregulated in the 12-OPDA-enriched *opr3-1* background. Expression of both genes was also enhanced in the single *atmyb60-1* and double *atmyb60-1 coi1-1* mutant combinations (Fig. 4i,j). This is in accordance with the overaccumulation of 12-OPDA in *atmyb60-1* and with the activation of a *COI1*-independent signaling pathway.

Taken together these results indicated that JA signaling was intact in *atmyb60-1* and that its constitutive reduction of stomatal opening was COI1-independent. We thus concluded that the stomatal defect depicted by *atmyb60-1* was primarily associated with the enhanced accumulation of 12-OPDA in guard cells, while the role of JA appeared dispensable.

### 12-OPDA induces stomatal closure cooperatively with ABA

**Figure 5:**
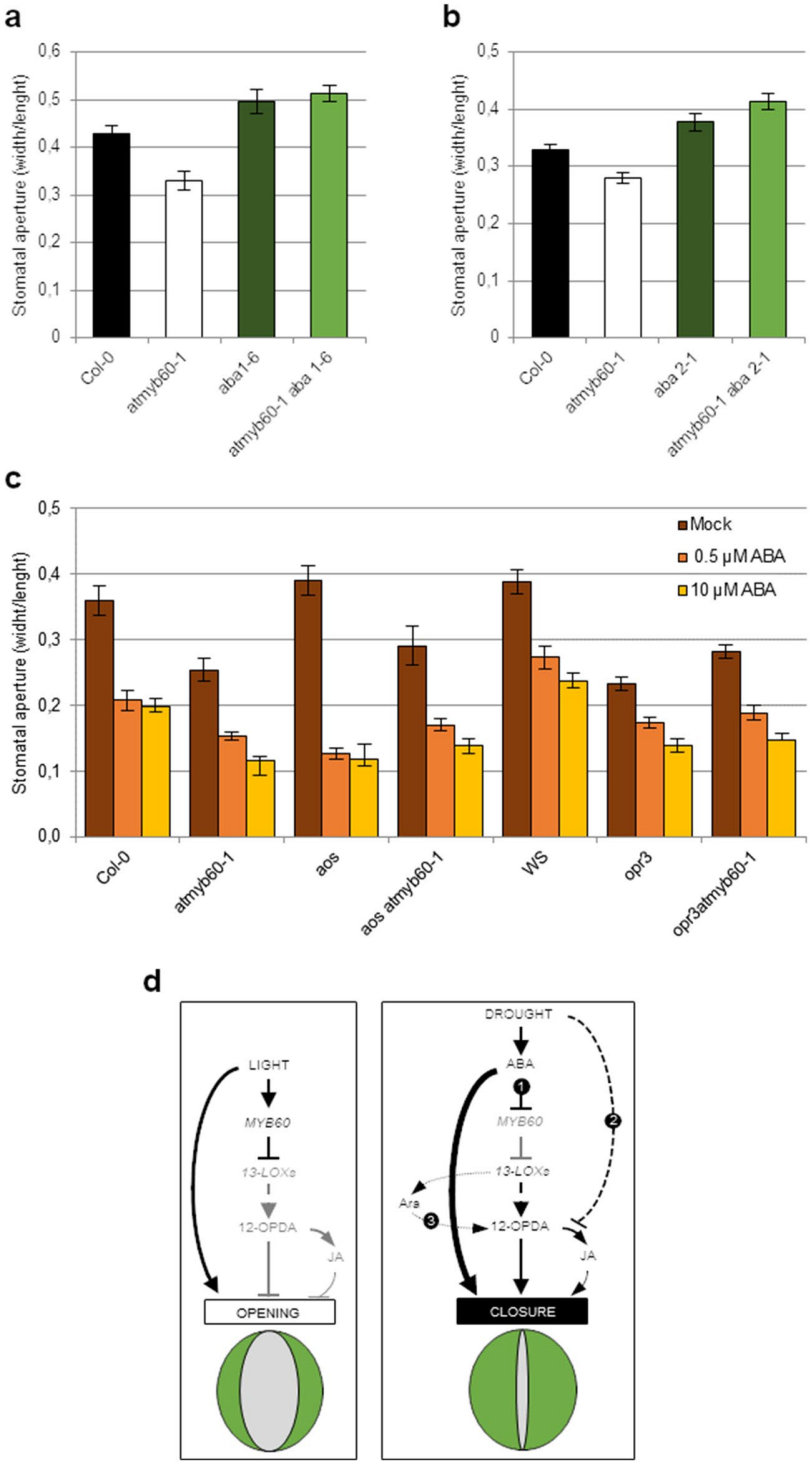
Oxylipin-ABA crosstalk in guard cells. (**a**,**b**) Stomatal aperture in Col-0, *atmyb60-1*, *aba1-6*, and *aba1-6 atmyb60-1* leaves (**a**) and in Col-0, *atmyb60-1*, *aba2-1*, and *aba2-1 atmyb60-1* leaves, after 4 h of exposure to light (**b**). (**c**) Stomatal aperture, in Col-0, *atmyb60-1*, *aos*, *aos atmyb60-1*, Ws, *opr3* and *opr3 atmyb60-1* leaves after 4 h of treatment with 0.5 or 10 µM ABA. (**d**) Working model of the *AtMYB60* function in guard cells. Under optimal growth conditions (left panel) *AtMYB60* restrains oxylipins production, by downregulating the expression of guard cell-related *13-LOXs*. Under drought (right panel) ABA plays a predominant role in triggering stomatal closure through the direct activation of ion channels (thick black line). Three interrelated pathways concur to enhance the level of free 12-OPDA in guard cells. (1) ABA indirectly promotes 12-OPDA synthesis by suppressing *AtMYB60* expression and releasing its negative effect on *13-LOXs* activity. (2) Drought uncouples the conversion of 12-OPDA to JA, favoring the accumulation of 12-OPDA over JA. (3) The release of 12-OPDA from Arabidopsides contributes to enhance the pool of free 12-OPDA. Accumulation of 12-OPDA promotes stomatal closure independently of JA and cooperatively with ABA.

Evidence indicates extensive crosstalk between oxylipins and ABA in several developmental and response pathways, including stomatal closure^28^. In particular, exogenous applications of 12-OPDA trigger stomatal closure in the wild type but not in the ABA-deficient *aba2-1* mutant, implying a role for ABA in oxylipin signaling in guard cells^6^. Addressing the ABA-oxylipins crosstalk in the context of the *atmyb60-1* mutation is of particular interest, considering that *AtMYB60* expression is rapidly downregulated in the presence of ABA^10, 12, 13^. To this task, we analyzed stomatal opening in the *atmyb60-1 aba1-6* and *atmyb60-1 aba2-1* double mutants. The *aba1-6* and *aba2-1* alleles impair early and late ABA biosynthesis, respectively, drastically reducing the accumulation of the hormone in the plant tissues^29, 30^. As expected, *aba1-6* and *aba2-1* showed increased stomatal opening compared with the wild type. Interestingly, opening of the stomatal pore was enhanced to the same extent in *atmyb60-1 aba1-6* and *atmyb60-1 aba2-1* as in the single *aba* mutants (Fig. 5a,b). This indicated that in the absence of ABA, the overaccumulation of 12-OPDA was insufficient to reduce stomatal opening in *atmyb60-1*.

Finally, we did not observe significant differences in ABA-induced stomatal closure between *atmy60-1* and the wild type or the *aos* and *opr3* biosynthetic mutants (Fig. 5c). This indicated that stomatal sensitivity to ABA was not altered by the lack of JA and 12-OPDA, as in the *aos* mutant, by the overaccumulation of 12-OPDA, as in the *opr3-1* background, or by the concurrent overaccumulation of JA and 12-OPDA, as in *atmyb60-1*.

Taken together our results confirmed that 12-OPDA modulates stomatal opening in an ABA-dependent manner and emphasized the cooperative action of ABA and oxylipins in mediating guard cell activity.

## Discussion

Despite the predominant role of ABA in regulating stomatal closure in response to water deficit, it is increasingly clear that other hormones, including oxylipins, contribute to modulate stomatal activity under stress. Oxylipins and ABA share several signaling components suggesting strong convergence between the two pathways^31^. Here we propose a role for *AtMYB60* as a transcriptional node in the crosstalk between oxylipins and ABA in stomata. Our working hypothesis entails three main concepts: (i) the occurrence of an autonomous oxylipin biosynthetic pathway in guard cells, (ii) the function of *AtMYB60* as a negative regulator of *13-LOXs* expression in guard cells, and (iii) the involvement of 12-OPDA in triggering stomatal closure under water stress.

We showed that guard cells accumulate substantial levels of JA, JA-Ile, 12-OPDA and Arabidopsides (Figs. 1d,e and 2b–k). It is well known that extensive root-to-shoot and cell-to-cell transport of JA and related oxylipins take place during stress responses^32^. This opens the possibility that the overaccumulation of 12-OPDA and JAs observed in *atmyb60-1* stomata could result from increased transport from other sites of synthesis. Nevertheless, the cellular specificity of *13-LOXs* expression favors the *in-situ* production of oxylipins in stomata. Gene profiling of LM-purified mesophyll and guard cells revealed the stomatal preference for *LOX4* and *LOX6*. Despite its predominant expression in the mesophyll, *LOX2* was highly expressed in guard cells, compared with other *13-LOXs* (Fig. 3a). This is of particular interest as *LOX2* is the major contributor to oxylipin synthesis in leaves upon wounding and osmotic stress, and it is responsible for channeling 12-OPDA and dnOPDA into Arabidopsides^33^. Evidence indicates that, the autonomous *de novo* synthesis of ABA is essential for stomatal closure in response to low air humidity or water deficit^34, 35^. Leaves often display a “patchy stomatal conductance”, with small groups of stomata behaving differently from those located in adjacent parts of the leaf. It is intriguing to speculate that, the independent synthesis of ABA and 12-OPDA in guard cells provides an additional layer of regulation fostering the spatial control of stomatal opening in response to local variations in the leaf water potential.

The cellular specificity of oxylipins biosynthesis evokes cell-specific regulatory mechanisms, including the control of transcription. Three lines of evidence support a role for AtMYB60 in modulating oxylipins synthesis in stomata through the regulation of *LOX2*, *LOX4* and *LOX6* expression. First, expression of these *13-LOXs* was constitutively enhanced in *atmyb60-1* guard cells compared with the wild type (Fig. 3a). Second, the transient co-expression of the AtMYB60 protein in tobacco leaves selectively downregulated the activity of *13-LOX* promoters (Supplementary Fig. 4a). Third, expression of individual *LOX2-*, *LOX4*- or *LOX6pro:GUS* constructs in the *atmyb60-1* background resulted in increased GUS activity in stomata (Fig. 3b–m, Supplementary Table 1). Interestingly, the *atmyb60-1* mutation did not affect *LOX3* expression (Fig. 3a). Differently from other *13-LOX*s, *LOX3* plays a unique role in JA-mediated responses to high salt conditions, which are not directly related to adjustments in stomatal aperture^36^. Similarly, *AtMYB60* did not regulate expression of *9-LOXs* (Fig. 3). 9-LOX-derived oxylipins are not directly involved in the plant response to drought as they mainly activate local defense and stomatal closure against pathogens^17^. This seems to exclude a role for *AtMYB60* in regulating stomatal oxylipins production in response to biotic stress.

AtMYB60 specificity for *LOX2*, *LOX4* or *LOX6*, does not necessarily imply the direct regulation of their transcription. As opposite to other members of the MYB family, a specific DNA binding motif for AtMYB60 has not been described^37^. A large-scale DNA affinity purification sequencing (DAP-seq) analysis of the Arabidopsis cistrome did not identify direct gene targets for AtMYB60^38^. This opens the possibility for an indirect regulation of *13-LOXs* expression, involving other transcription factors downstream of AtMYB60. Further studies will be required to address the molecular details of the AtMYB60 mode of action.

Increased expression of *13-LOXs* in *atmyb60-1* guard cells resulted in the overaccumulation of 12-OPDA, JAs and Ara (Figs. 1d,e, 2b–k). The involvement of these oxylipins in mediating stomatal activity is still controversial. Application of 10 μM Me-JA was shown to reduce stomatal aperture in wild type L*er* plants^39^. By contrast, other studies reported that concentrations of Me-JA up to 100 μM were ineffective in reducing stomatal aperture in Col-0^6, 17^. Gimenez-Ibanez and colleagues demonstrated that the guard cell-specific COI1-JAZ2-dependent JA signalling is hijacked by bacteria to promote stomatal opening during infection^40^. Similarly, the *Pseudomonas syringae* effector protein AvrB induces stomatal opening through a guard cell-related JA pathway^41^. These findings imply the involvement of the endogenous JA signalling in promoting stomatal opening rather than stomatal closure. At the opposite, evidence indicates 12-OPDA as drought-responsive modulator of stomatal closure, acting in cooperation with ABA. 12-OPDA efficiently induces closure of the stomatal pore at concentrations significantly lower than Me-JA and the overaccumulation of 12-OPDA in the *opr3-1* mutant results in enhanced stomatal closure^6, 17^. Most importantly, it has been shown that drought selectively induces the accumulation of 12-OPDA in leaf tissues, while JA remains at physiological levels^6^. Consistent with these findings, our results suggested that the constitutive reduction of stomatal opening in *atmyb60-1*was mainly associated with the overaccumulation of 12-OPDA rather than JA in guard cells.

Here we propose a working model integrating previously published data with novel evidence. It has been shown that *AtMYB60* is actively expressed in guard cells under conditions promoting stomatal opening (e.g. light)^10^. According to our model, under favorable conditions, *AtMYB60* downregulates the expression of *13-LOXs*. This, in turns, reduces the pool of 12-OPDA in stomata, thereby favoring the opening of the pore (Fig. 5d, left panel). Upon perception of water deficit, accumulation of ABA plays a major role in triggering stomatal closure by directly activating the efflux of anions and potassium through plasma membrane ion channels (Fig. 5d, right panel). Under drought, ABA induces the rapid downregulation of *AtMYB60* expression^10–13^, which results in the activation of the guard cell-related *LOX2*, *LOX4* and *LOX6* lipoxygenases. According to our hypothesis, increase in 13-LOX activity leads to the accumulation of 12-OPDA which promotes stomatal closure cooperatively with ABA.

In addition to triggering ABA production, drought generates a yet to be identified signal which uncouples the conversion of 12-OPDA to JA, thus favoring the selective accumulation of 12-OPDA^6^ (Fig. 5d, right panel). In Arabidopsis, the major fraction of 12-OPDA is esterified to galactolipids to produce Arabidopsides^16^. Remarkably, *atmyb60-1* stomata accumulated increased amounts of Ara-A, -B and -D relatively to the wild type (Fig. 2f–k). Even if the functional significance of Arabidopsides is still debatable, evidence indicates that they act as storage compounds of 12-OPDA, which at the occurrence can be rapidly mobilized^42^. Upon perception of drought, the release of 12-OPDA from Arabidopsides could contribute to the rapid accumulation of free 12-OPDA, and thus to stomatal closure (Fig. 5d, right panel).

It has been suggested that the loss of the AtMYB60 function constitutively activates a stress response signal which results in long-term beneficial effects under stress^10^. Our study identified such a signal with the enhanced accumulation of oxylipins in guard cells, providing a mechanistic explanation for the superior drought resistance exhibited by the *atmyb60-1* mutant. The proposed involvement of the oxylipin pathway in modulating stomatal opening is consistent with the finding that, even if ABA is clearly the most effective hormone in reducing stomatal aperture, its efficacy is enhanced when ABA and 12-OPDA are co-applied^6^. The stress-activated synthesis of oxylipins in guard cells could also play an additional and, perhaps more relevant role, in the so called “after drought effect”. Following stress release, the reopening of stomatal pores is usually very slow and stomatal conductance hardly reaches the levels of unstressed plants. The *AtMYB60*-mediated accumulation of 12-OPDA could provide an enduring signal to sustain stomatal closure during rewatering, favouring the rehydration of the plant tissues and preventing premature stomatal opening, even when ABA levels decline.

Considering the strong conservation of the *AtMYB60* regulatory network between Arabidopsis and distantly related species, including tobacco, tomato and grape^13, 43, 44^, engineering of the *AtMYB60*-dependent oxylipin biosynthetic pathway could provide an attractive strategy to enhancing crop survival and productivity under stress.

## Methods

### Plant material and plant growth

This study employed two wild type Arabidopsis ecotypes, Columbia (Col-0) and Wassilewskija (Ws), obtained from NASC (N1092 and N1602, respectively). The *atmyb60-1* mutant was originally selected by Cominelli et al.^10^. The *aba1-6* (N3772) and *aba2-1* (N156) mutants were obtained from NASC. The *aos* and *opr3* mutants were kindly provided by K. Dehesh, and the *coi1-1* and *coi1-30* alleles by A. Chini. All the mutants are in the Col-0 background with the exception of *opr3* (Ws). Double mutant combinations were generated by crossing and selected as described in Supplementary table 2. Identification of the plant material employed in this study was performed by F. S. and L. S. Seedlings used in GUS and qPCR experiments were grown in vitro. Seeds were surface sterilized with 100% ethanol followed by 1% NaClO, washed with sterile water and plated on 1.5% sucrose MS medium, (0.8% agar, pH 5.8). Plates were stratified at 4 °C for 4 days in the dark before moving into growth room at 22 °C under long-day conditions (16-h light/8-h dark; 160 μmol m^−2^ s^−1^). Plants used for metabolite profiling, laser-microdissection and stomatal assays were grown in soil in a semi-controlled greenhouse (temperature 19–23 °C, relative humidity 65%). Natural light was supplemented by metal halide lamps when inferior to 150 μmol m^−2^ s^−1^ in a long day photo cycle. For each experiment, rosette leaves were collected in the morning, following 4 h of exposure to light. Greenhouses experiments were performed at the Botanical Garden “Città Studi”, in compliance with the institutional, national, and international guidelines and legislation.

### Leaf blending

3-week-old Col-0 and *atmyb60-*1 rosettes grown in soil were excised and blended according to Bauer et al.^34^. In brief, leaves were whisked with a blender in ice-cold deionized water with crushed ice for 1 min and then filtered through a 210 μm nylon net. After three rounds of whisking, the resulting epidermal fractions were frozen in liquid nitrogen and stored at -80 °C. Six independent biological replicas were prepared for metabolite profiling, each consisting of a pool of 15–20 rosettes.

### Metabolite profiling

Samples for hormone and lipidome analysis were prepared according to Salem et al.^45^. In short, plant ground material was extracted with a mixture of MTBE:MeOH (3:1, v:v) and separated into two aliquots. The aliquot for lipidome analysis was phase separated by adding a mixture of H_2_O:MeOH (3:1, v:v) to the MTBE:MeOH extract. The upper lipophilic phase was collected, dried and re-suspended in ACN:Isopropanol (7:3, v:v) before analysis in a UHPLC-MS system. The aliquot used for hormone profiling was phase separated using aqueous 0.1% hydrochloric acid solution. The upper lipophilic phase was collected, dried and re-suspended in MeOH:H_2_O (1:1, v:v). Lipidomics samples were separated on a Acquity UPLC (Waters) system using an RP8 column and analyzed on a LTQ Orbitrap XL (ThermoFisher Scientific) mass spectrometer following the methodology described by Hummel et al.^46^. Hormone samples were separated on a Acquity UPLC (Waters) system using an RP18 column and analyzed on a QTRAP4000 (ABSciex) mass spectrometer following the methodology described by Salem et al.^45^.

### Laser capture microdissection of leaf tissues and RNA purification

Leaf tissues from Col-0 and *atmyb60-1* plants were prepared according to Kerk et al.^47^ and microdissected using the Pix‐Cell II LCM system (Arcturus Engineering). RNA from LCM‐harvested cells was prepared using the PicoPure kit (Arcturus Engineering), and reverse‐transcribed using the Superscript™ II reverse transcriptase (Invitrogen).

### Plasmid construction

The *LOX6*_*pro*_*:GUS* line was kindly provided by E.E. Farmer. Construction of the *LOX2*_*pro*_*:GUS*, *LOX3*_*pro*_*:GUS*, and *LOX4*_*pro*_*:GUS* fusion has been previously described^48^. For the *LOX1*_*pro*_*:GUS* construct a 2414 bp genomic fragment upstream of the *LOX1* start codon was amplified with the primers pLOX1-F1 (5′-CACCATCTTGCCTTGGCCACGTTAAT-3′) and pLOX1-R1 (5′-TTGATTCACTCTGCTCTC TCT CTA ATT-3′) and inserted, by Gateway cloning into the binary vector pBGWFS7 (Invitrogen). For the cloning of the *LOX5* promoter a 2321 bp genomic fragment was amplified with the primers pLOX5-F1 (5′-CACCGAAGATTAAGTTATGGATGGAAACAAGGAT-3′) and pLOX5-R1 (5′-TGCAGAATTTTCTCTGAGTAAGAATCAAGA-3′) and inserted into pBGWFS7. Constructs were transformed into Col-0 by the floral-dip method. Lines with single insertion were identified base on segregation analysis of BASTA-resistance.

### GUS staining

Whole seedlings or excised leaves were vacuum‐infiltrated in the staining solution (50 mM sodium phosphate buffer, pH 7, 0.1% Triton‐X100, 0.5 mg ml^− 1^ X‐glucoronic acid and 0.5 mM FeCN) and incubated at 37 °C for 24 h. Tissue were cleared with 70% ethanol and analyzed using an Olympus SZX12 stereomicroscope or a Zeiss Imager2 microscope.

### Transient expression in *N. benthamiana*

Leaves from 5-week-old *Nicotiana benthamiana* plants were Agroinfiltrated as described^49^. In total 20 leaves (5 leaves from four different plants) were co-infiltrated with individual *LOX*_*pro*_*:GUS* constructs and with the *CaMV35S:AtMYB60* plasmid or with a control empty vector. Leaf samples were collected at 48 h after the Agroinfiltration and GUS expression was analyzed by qPCR with the primers pGUS-F (5′-TACGGCAAAGTGTGGGTCAATAATCA-3′) and pGUS-R (5′-CAGGTGTTCGGCGTGGTGTAGAG-3′) and normalized to expression of the *Elongation factor 1a* (*EF-1a*) gene, using the primers pEF1-F (5′-AGCTTTACCTCCCAAGTATC-3′) and pEF1-R (5′-AGAACGCCTGTCAATCTTGG-3′).

### Quantification of mRNA expression

Total RNA was isolated with TRIzol reagent following the manufacturer’s instructions (Life Technologies). First-strand cDNA was synthesized from 1 µg of RNA using the SuperScript VILO cDNA Synthesis Kit (Invitrogen). Quantification of the relative transcript abundance was performed as described^50^. The reference *AtACTIN2* gene (At3g18780) was used for normalization. Sequences of the primers used in the qPCR experiments are listed in Supplementary Table 3. Each amplification was performed on three independent biological replicates.

### Stomatal opening and stomatal conductance

Epidermal strips prepared from dark-adapted plants were incubated in 30 mM KCl, 10 mM MES-KOH, pH 6.5, at 22 °C, and exposed to light (200 µmol m^−2^ s^−1^) for 4 h. Indicated concentrations of MeJA, ABA (SIGMA-Aldrich) or 12-OPDA (Cayman Chemicals) were added to the solution to assay for stomatal closing. Images of stomata were taken with a Zeiss Imager2 microscope fitted with a digital camera and analyzed with the ImageJ program (https://imagej.net/) to measure width and length of individual stomatal pores. Stomatal conductance was measured on individual leaves from 3-week-old plants grown in soil, using a portable SC-1 porometer (Decagon Devices).

## Supplementary Information

Supplementary Information.
