# Supplementary material for: The AtMYB60 transcription factor regulates stomatal opening by modulating oxylipin synthesis in guard cells
Source: Sci Rep. 2022 Jan 11;12:533. doi: 10.1038/s41598-021-04433-y (PMC8752683; doi:10.1038/s41598-021-04433-y)
Supplement: Supplementary file 1 — Supplementary Information. [file 41598_2021_4433_MOESM1_ESM.pdf]

Supplementary Material

The AtMYB60 transcription factor regulates stomatal opening by modulating oxylipin synthesis in guard cells

Fabio Simeoni, Aleksandra Skirycz, Laura Simoni, Giulia Castorina, Leonardo Perez de Souza, Alisdair R. Fernie, Saleh Alseekh, Patrick Giavalisco, Lucio Conti, Chiara Tonelli and Massimo Galbiati.

Supplementary Figure 1

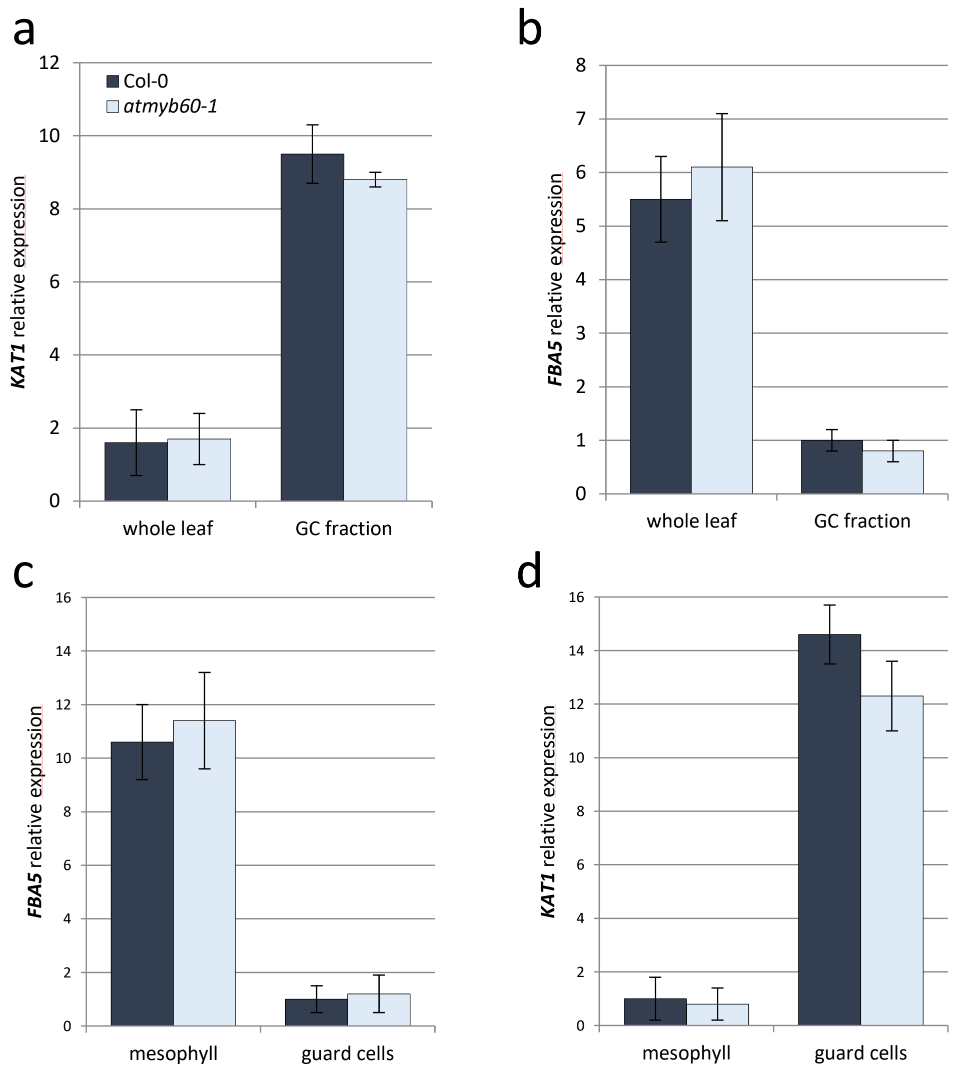

Supplementary Fig. 1: tissue specificity of the blending- or LM-derived samples employed in this study. a-d, qPCR analysis of the mesophyll-specific gene *Fructose-Bisphosphate Aldolase 5* (*FBA5*, At4g26530) (Yang et al., 2008) (**a**, **c**) and of the guard cell-specific gene *Potassium Channel in Arabidopsis thaliana 1* (*KAT1*, At5g46240) (Schachtman et al., 1992) (**b**, **d**). Analyses were performed on whole leaves and blending-derived epidermal fragments enriched in guard cells (**a**, **b**), or in LM-purified mesophyll cells and guard cells (**c**, **d**). Relative gene expression was normalized to the expression of the *AtACTIN2* gene.

Supplementary Figure 2

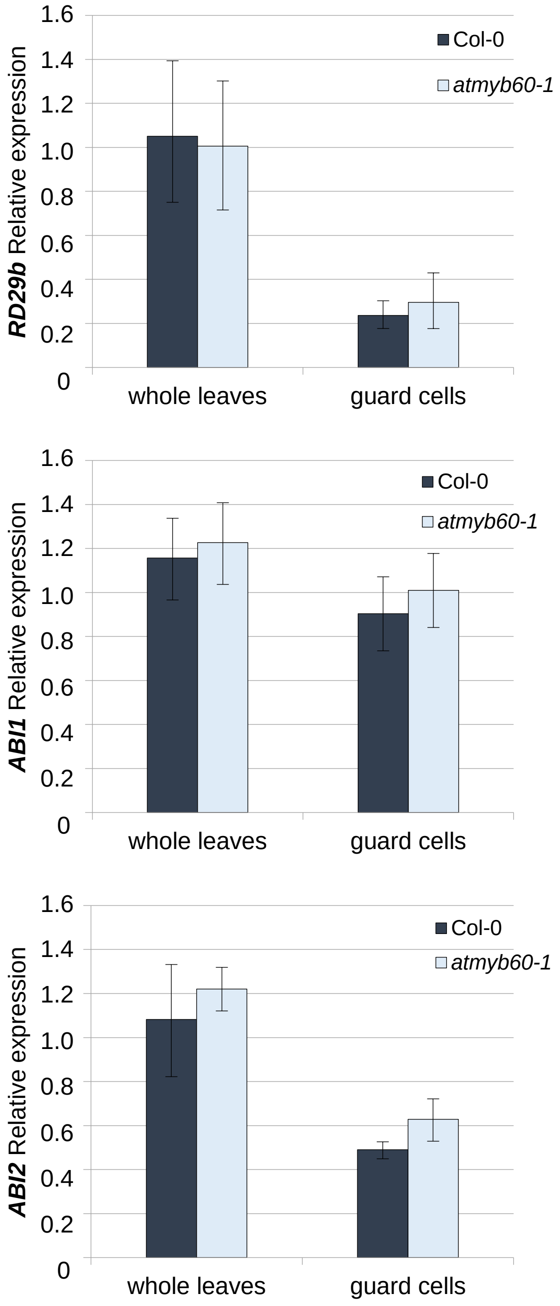

Supplementary Fig.2: expression of ABA-induced genes in whole leaves and guard cells from wild type and *atmyb60-1* plants. qPCR analyses of *RESPONSIVE TO DESICCATION 29B* (*rd29B*, At5g52300) (**a**), *ABA INSENSITIVE1* (*ABI1*, AT4G26080) (**b**) and *ABA INSENSITIVE2* (*ABI2*, At5g57050) (**c**) were performed on whole leaves, and epidermal fragments enriched in guard cells. Relative gene expression was normalized to the expression of the *AtACTIN2* gene (At3g18780).

Supplementary Figure 3

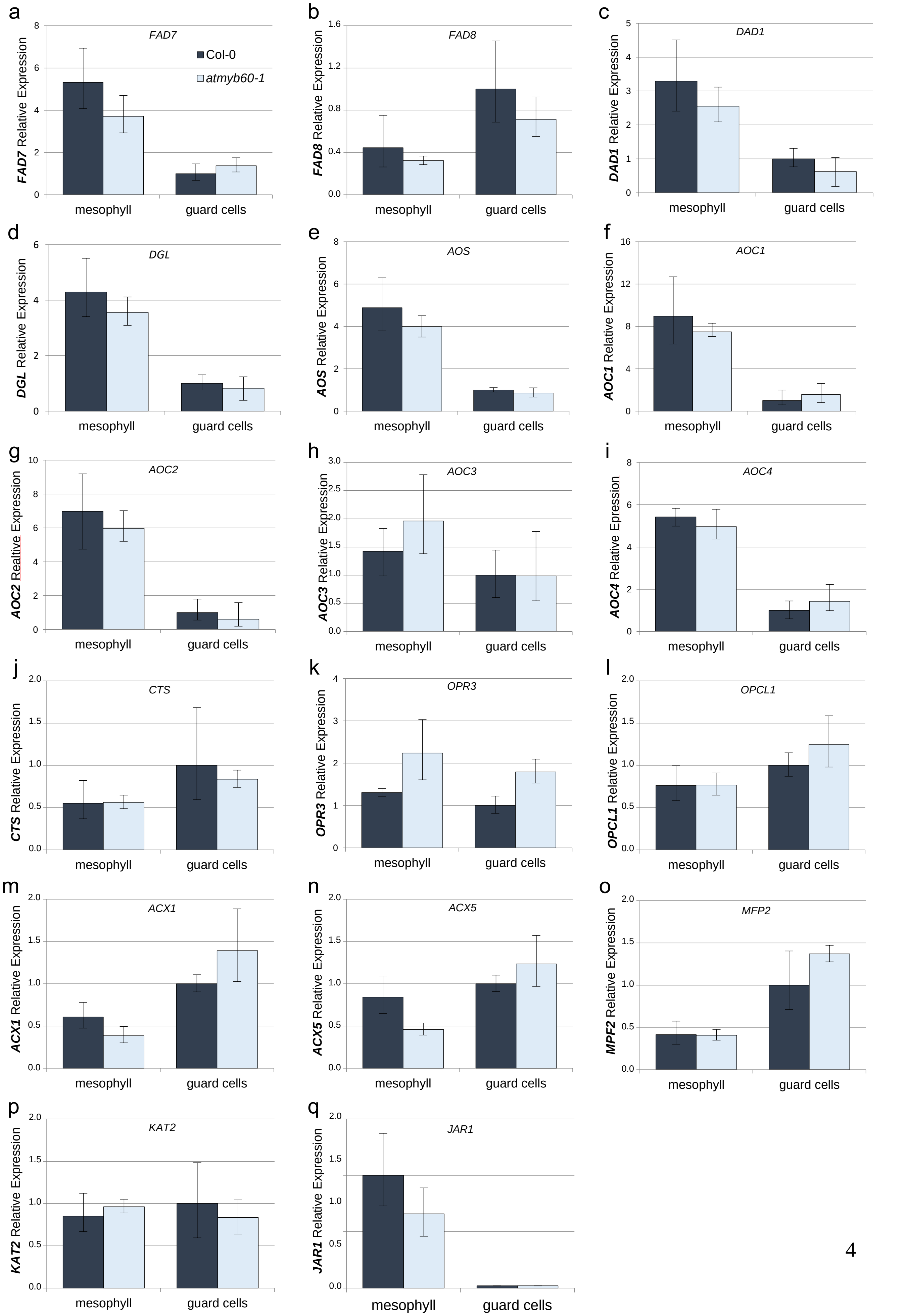

Supplementary Fig. 3: expression of JA biosynthetic genes in LM-purified wild type or *atmyb60-1* mesophyll and guard cells. Expression of individual genes was investigated by qPCR analysis of mesophyll and guard cells laser-microdissected from wild type or *atmyb60-1* leaves. Relative gene expression was normalized to the expression of the *AtACTIN2* gene. **a**-**b**, expression of *Fatty Acid Desaturase7* and *-8*. **c**-**d**, expression of the lipases *Defective in Anther Dehiscence1* and *Dongle*. **e**, expression of A*llene Oxide Synthase*. **f**-**i**, expression of the four Arabidopsis *Allene Oxide Cyclases*. **j**, expression of *Comatose1*. **k**, expression of *Opda Reductase3*. **l**, expression of *OPC-8:0 CoA Ligase1*. **m**-**n**, expression of *ACYL-CoA Oxidase1* and *-5*. **o**, expression of *Multifunctional Protein2*. **p**, expression of *L-3-Ketoacyl CoA Thiolase2*. **p**, expression of *Jasmonate Resistant1*. Expression of the six Arabidopsis *Lipoxygenases* gene (*LOX1-6*) is provided in Fig. 3a in the main text.

Supplementary Figure 4

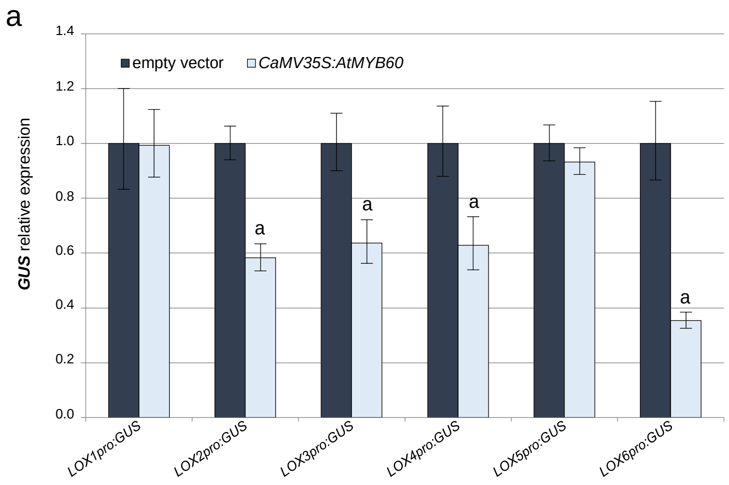

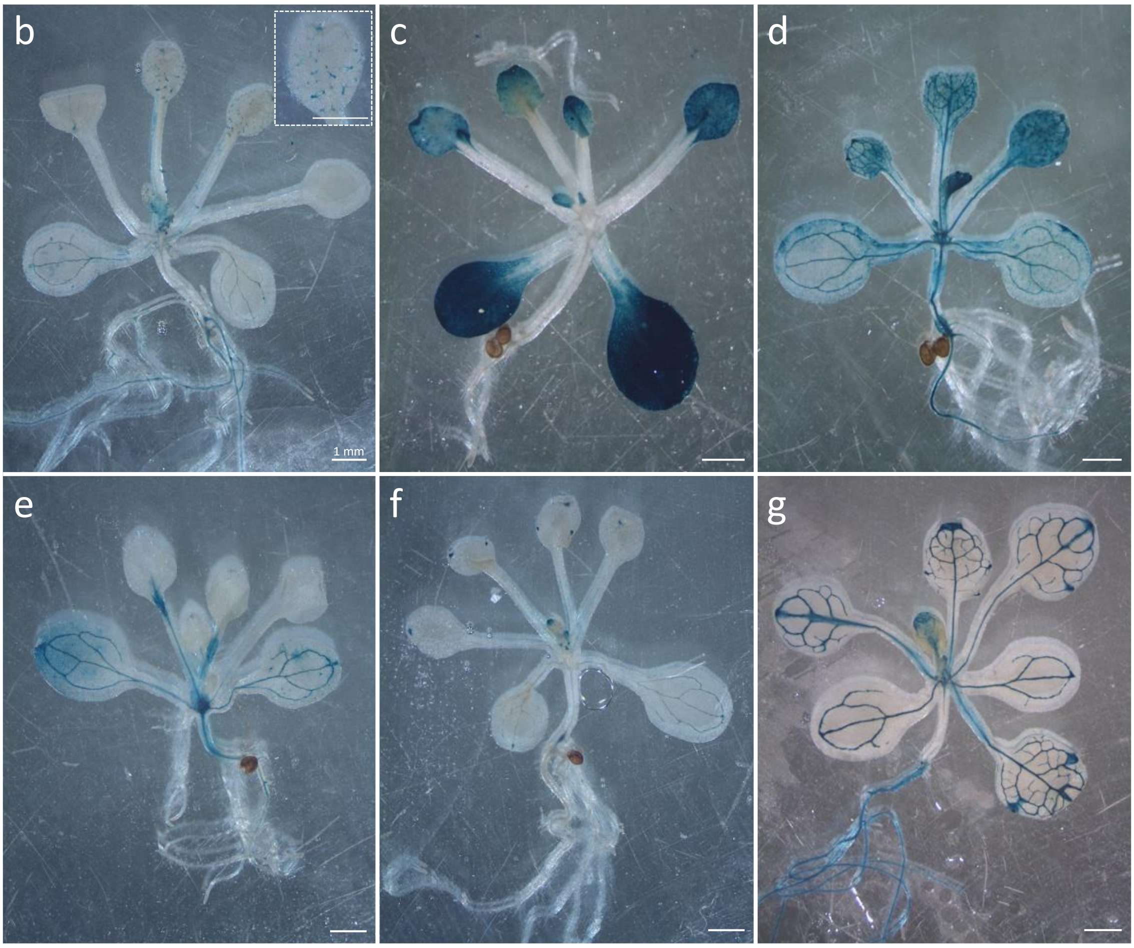

Supplementary Fig. 4: Analysis of the LOX promoter activities. a, transient expression assay in *N. benthamiana*. Leaves were co-transfected with the activating construct *CaMV35S:AtMYB60* and individual LOX_{pro}*:GUS* target constructs. The *CaMV35S* empty vector was used as a negative control. *GUS* expression was investigated at 48 hours from the co-transfection by qPCR and normalized to the tobacco *EF-1a* gene. Data are means of four biological replicates ± standard errors. “a” indicates significant differences relatively to the empty vector control ($P<0.01$, *t*-test). **b-g,** GUS expression patterns in the Col-0 *LOXpro:GUS* lines. 15-day-old seedlings were incubated in the GUS staining solution for 24 hours and cleared with 70% ethanol. 15 to 20 independent lines were analyzed for each LOX_{pro}*:GUS* construct. Representative images of individual transgenic lines are provided. **b**, $LOX1_{pro}$*:GUS* line. The boxed image is a magnified view of a developing leaf with GUS-stained trichomes. **c**, $LOX2_{pro}$*:GUS* line, showing strong GUS expression in the mesophyll. **d**, $LOX3_{pro}$*:GUS* line, showing strong activity in the vasculature and weaker expression in the mesophyll. **e**, $LOX4_{pro}$*:GUS* line, displaying intense staining of vascular tissues. **f**, $LOX5_{pro}$*:GUS* line, showing moderate staining of the vasculature and intense staining of hydathodes. **g**, $LOX6_{pro}$*:GUS* line, with strong GUS activity in the vascular system. Scale bars represent 1 mm.

Supplementary Figure 5

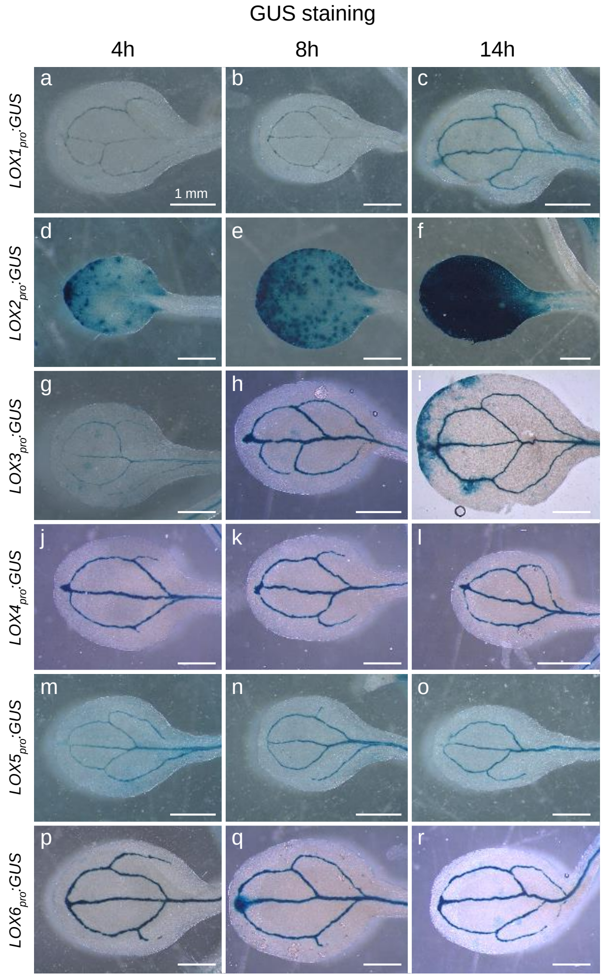

Supplementary Fig. 5: Kinetic of GUS activity in the *LOX*$_{pro}$*:GUS* lines. a-r, Representative images of cotyledons or leaves from individual transgenic lines. 10-day-old seedlings were incubated in the GUS staining solution for 4, 8 or 14 hours and cleared with 70% ethanol. No GUS activity was detected in stomata from any of the *LOX*$_{pro}$*:GUS* when stained for up to 14 hours. Scale bars represent 1 mm.

Supplementary Table 1: number of GUS-positive stomata identified in wild type (Col-0) and *atmyb60-1* transgenic lines expressing individual $LOX_{pro}:GUS$ constructs. Developing leaves from 15-day-old seedlings were GUS-stained for 24 hours and cleared in 70% ethanol. Four areas of 60 x 40 μm from the central part of the leaf were imaged with an optical microscope. Five selected leaves were analysed for each $LOX_{pro}:GUS$ lines, for a total of 20 images/line corresponding to an overall leaf area of $48*10^3$ μm^2. Figures in Table 1 refer to the total number of GUS-positive or GUS-negative stomata spotted in the total leaf area analyzed.

line	Col-0		*atmyb60-1*	
	No. of GUS-positive stomata	No. of GUS-negative stomata	No. of GUS-positive stomata	No. of GUS-negative stomata
$LOX1_{pro}:GUS$	128 (16.16%)	664 (83.83%)	118 (14.79%)	680 (85.21%)
$LOX2_{pro}:GUS$	68 (9.88%)	620 (90.11%)	160 (19.61%)	656 (80.39%)
$LOX3_{pro}:GUS$	2 (0.29%)	681 (33.33.0%)	2 (0.28%)	694 (99.72%)
$LOX4_{pro}:GUS$	64 (9.03%)	644 (90.96%)	168 (19.99%)	673 (80.01%)
$LOX5_{pro}:GUS$	1 (0.15%)	649 (99.84%)	2 (0.31%)	644 (99.69%)
$LOX6_{pro}:GUS$	56 (7.42%)	699 (92.58%)	138 (17.76%)	639 (82.24%)

Supplementary Table 2: selection methods employed for the constitution of the double mutant combinations.

Allele	Selection methods			Reference
	resistance	phenotype	PCR	
atmyb60-1	BASTA		p60A 5'-GCAGATCCAAAAGAGGTTGTCAAGA-3'<br>p558 5'-CTCAAGCACACTTCAATTTCCTATAACG-3'	Cominelli, 2005
aba1-6			MR115 5'-GCTCGGAGTAAAGGCGGCGA-3'<br>MR116 5'- CAGGAAGTCCCCGTGACGCC-3'	Audran, 2001
aba2-1			LCM108 5'-GCGATGACTCGCGGTACATAT-3'<br>LCM119 5'- ACATGATAAATTGGCGGACA-3'	González-Guzmán, 2002
aos	kanamycin	male sterile		Savchenko, 2014
opr3	kanamycin	male sterile		Savchenko, 2014
coi1-1	coronatine	male sterile		Xie, 1998
coi1-30	coronatine	male sterile		Yang, 2012

Supplementary Table 3: primers used in the qPCR experiments performed in this study.

Gene	Gene ID	Forward (5'-3')	Reverse (5'-3')
Actin2	AT3G18780	CTCTCCCGCTATGTATGTCGCCA	GTGAGACACCATCACCAG
AtMYB60	AT1G08810	CATGAAGATGGTGATCATGAGG	TTCCATTTGACCCCCAGTAG
FAD7	AT3G11170	CCATGTTTTGGGCTCTCTTTGTT	CCACACTGTTCAACTTCGGATCA
FAD8	AT5G05580	GCCTCTAACCCTAAACCCA	CGGGAATTGAGAAGAGAAGAA
DAD1	AT2G44810	TCGGTAAGGAGCTTCGGCTGAG	CTGAATGGACACGTGGAGCTCAC
DGL	AT1G05800	TCGAATCCGGGTTCTTAGGTTTATAC	CATACTATGTCCCGCAAGTGTTATGC
LOX1	AT1G55020	ACTCTTCGTCTTGTAAGCTCTG	GTGACCTTGAAAGCGGATTCG
LOX2	AT3G45140	TTTGCTCGCCAGACACTTG	GGGATCACCATAAACGGCC
LOX3	AT1G17420	ACGTTGTCGTACTGGTCGCC	GTCTCGTGGCACATACATAGGTAATG
LOX4	AT1G72520	AAGGTCTCCCTGCTGATCTCAT	AAGCCCATGTGGTTGTGTTG
LOX5	AT3G22400	GGCAAAACCGGCCGTAAAT	CGTCCCTTGGCACGTATATGTT
LOX6	AT1G67560	GGCGATTTGACATGGAAGGA	ACAAGCCTCACGCCACATTC
AOS	AT5G42650	CGGGCGGGTCATCAAGTTC	GCTCCCATCGTGAGTTCTCC
AOC1	AT3G25760	CCCAGACCAAGCAAAGTTCAAG	TCTCCGAGACCAAACCTAAAGC
AOC2	AT1G13280	ATCACTCACCACATAAAGTAAAAGTCTC	CGATGAAATTGTTGATTACATGAAAGATTG
AOC3	AT3G25780	CTTTCTTCTGGAATTGGGGC	CTTTCTTCTGGAATTGGGGC
AOC4	AT1G13280	AATGTGTCCCGTCCCTATAAGC	AATGTGTCCCGTCCCTATAAGC
CTS	AT4G39850	GAGATTAGGCATGGCACGTT	GTCGCATTTGTGCATTCATC
OPR3	AT2G06050	GGACGCAACTGATTCTGACCCAC	GGACGCAACTGATTCTGACCCAC
OPCL1	AT1G20510	TTTATTGTTTGTGCTGTAGACCTGTAG	GCCATTTATGTGTGTAATCTTCTGTG
ACX1	AT4G16760	ACGGATGTAACTGAAAGACTGGC	GTGGTGGTGAGAGACTTCAATCCTG
ACX5	AT5G65110	TGTTCACGCCTTCATTGTTCCG	TCAACGCACCATTATCCACTCCATT
MPF	AT3G06860	ACACTCATCAATCCTCCCGTCA	CATCATTCCTGCTCAAGGCCT
KAT5	AT5G48880	GGGGAGATAATCGAGGGCTG	GGGGAGATAATCGAGGGCTG
JAR1	AT2G46370	GTTCAATACGTCTTTGCCGTC	GCAGCTTCGACATTGCAGTA
VSP1	AT5G24780	GCATCTCATACTCAAGCCAAACG	TCCTCAACCAAATCAGCCCA
VSP2	AT5G24770	GTTAGGGACCGGAGCATCAA	TCAATCCCGAGCTCTATGATGTT
JAZ10	AT5G13220	ACCAACAACGCTCCTAAGCC	GGAGTAGACGGAACCGAAC
JAZ12	AT5T20900	GCTGCACAGCCATTTCCTA	CTCGAGGAATCGTTGAAGC
HSP20	AT1G52560	CGACGATCATGGCTACTTCAC	TAGCCCTGGAACCTCGTATCT
ZAT12	AT5G59820	CCACGGTGACTACGTTGAAGA	TAAACTGTTCTTCCAAGCTCCA
FBA5	AT4G26530	GTGCACGTTGCCAGGAGTATT	GAGGACTGCACGCCATTTTG
KAT1	AT5G46240	ACAACGGAAGCGAACTAGGA	AGCGAACAAAGTGACCGAAATG
RD29b	AT5G52300	TGACTCCGGTTTACGAAAAAGT	GTCTCCTTCACTCCACTTCCAC
ABI1	AT4G26080	TGAGATGGCAAGGAAGCGGATT	TGCCTCAGTTCAAGGGTTTGCT
ABI2	AT5G57050	GGAGTGACTTCGATTTGTGGTAGACG	GTCAAAGCCAGATGCATCCTCTCACG

Bibliography

Audran, C. *et al.* Localization and expression of zeaxanthin epoxidase mRNA in Arabidopsis in response to drought stress and during seed development. *Aust. J. Plant Physiol.* **28**, 1161–1173 (2001).

Cominelli, E. *et al.* A guard-cell-specific MYB transcription factor regulates stomatal movements and plant drought tolerance. *Curr. Biol.* **15**, 1196–1200 (2005).

González-Guzmán, M. *et al.* The short-chain alcohol dehydrogenase ABA2 catalyzes the conversion of xanthoxin to abscisic aldehyde. *Plant Cell* **14**, 1833–1846 (2002).

Savchenko, T. *et al.* Functional convergence of oxylipin and abscisic acid pathways controls stomatal closure in response to drought. *Plant Physiol.* **164**, 1151–1160 (2014).

Schachtman D, Schroeder J, Lucas W, Anderson J, G. R. S. Expression of an inward-rectifying potassium channel by the Arabidopsis KAT1 cDNA. *Science* **258**, 1654–8. (1992).

Xie, D. X., Feys, B. F., James, S., Nieto-Rostro, M. & Turner, J. G. COI1: An Arabidopsis gene required for jasmonate-regulated defense and fertility. *Science* **280**, 1091–1094 (1998).

Yang, D. L. *et al.* Plant hormone jasmonate prioritizes defense over growth by interfering with gibberellin signaling cascade. *Proc. Natl. Acad. Sci. U. S. A.* **109**, (2012).

Yang, Y., Costa, A., Leonhardt, N., Siegel, R. S. & Schroeder, J. I. Isolation of a strong Arabidopsis guard cell promoter and its potential as a research tool. *Plant Methods* **4**, 1–15 (2008).
